# Second-Order Kinetic Rate Coefficients for the Aqueous-Phase Sulfate Radical (SO_4_^•–^) Oxidation of Some Atmospherically Relevant Organic Compounds

**DOI:** 10.1021/acs.jpca.2c04964

**Published:** 2022-09-07

**Authors:** Lillian N. Tran, Karizza A. Abellar, James D. Cope, Tran B. Nguyen

**Affiliations:** † Department of Environmental Toxicology, University of California Davis, Davis, California 95616, United States; ‡ Department of Chemistry, University of California Davis, Davis, California 95616, United States

## Abstract

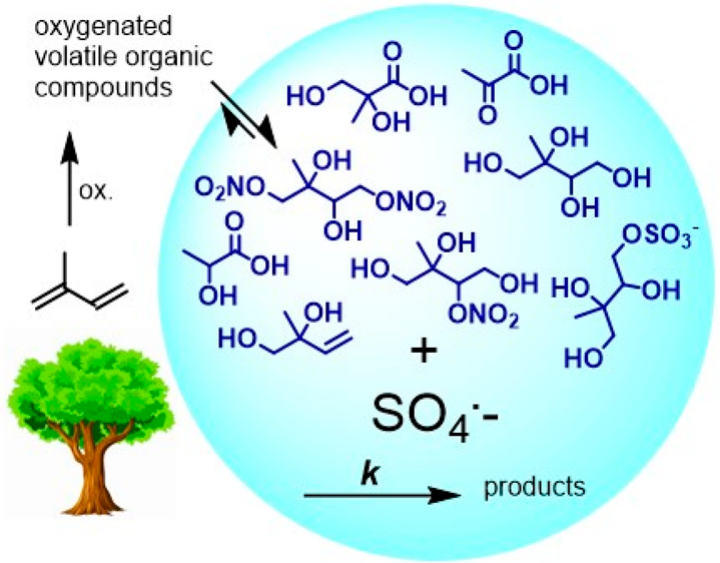

The sulfate anion radical (SO_4_^•–^) is a reactive oxidant formed in the autoxidation chain of sulfur dioxide, among other sources. Recently, new formation pathways toward SO_4_^•–^ and other reactive sulfur species have been reported. This work investigated the second-order rate coefficients for the aqueous SO_4_^•–^ oxidation of the following important organic aerosol compounds (*k*_SO4_): 2-methyltetrol, 2-methyl-1,2,3-trihydroxy-4-sulfate, 2-methyl-1,2-dihydroxy-3-sulfate, 1,2-dihydroxyisoprene, 2-methyl-2,3-dihydroxy-1,4-dinitrate, 2-methyl-1,2,4-trihydroxy-3-nitrate, 2-methylglyceric acid, 2-methylglycerate, lactic acid, lactate, pyruvic acid, pyruvate. The rate coefficients of the unknowns were determined against that of a reference in pure water in a temperature range of 298–322 K. The decays of each reagent were measured with nuclear magnetic resonance (NMR) and high-performance liquid chromatography–high-resolution mass spectrometry (HPLC-HRMS). Incorporating additional SO_4_^•–^ reactions into models may aid in the understanding of organosulfate formation, radical propagation, and aerosol mass sinks.

## Introduction

A wide variety of water-soluble condensed-phase compounds are formed during the oxidation of reactive carbon emissions in the atmosphere.^1−3^ The oxidation of reactive carbon emissions in the aqueous phases of aerosols, fogs, and clouds initiated by hydroxyl (OH) radicals, nitrate (NO_3_) radicals, and the sulfate anion radical (SO_4_^•–^) has been reviewed previously (refs (4−6) and references therein). SO_4_^•–^ radicals have been recognized as a potentially important source of surface-active organosulfates (OSs) in the environment,^7−10^ which can modify aerosol cloud interactions.^11,12^ A large variety of OSs have been identified in both field and laboratory studies, several of which are proposed to only be formed from SO_4_^•–^ radicals.^9,13−17^ The SO_4_^•–^ radical is thought to be formed primarily in the autoxidation of SO_2_; however, it can also be formed from the activation of sulfate and bisulfate anions with OH, NO_3_,^4^ and to a small extent ferric ion complexes.^18,19^ Although the atmospheric concentration of the SO_4_^•–^ radical has not been directly measured, it has been estimated between 9.1 × 10^–13^ and 5.5 × 10^–17^ M, assuming known sources.^4^ New mechanisms of reactive sulfur species relevant to sulfate aerosols have been recently reported, including SO_4_^•–^ formation from irradiated sulfate aerosols,^20^ OH-initiated oxidation of organosulfates,^21^ and autocatalysis of the SO_4_^2–^ anion in the presence of phenols.^22^ The formation of reactive sulfur was also reported from the interfacial redox of ammonium sulfate aerosol particles.^23^ Thus, the chemistry and environmental source calculations of SO_4_^•–^ and other sulfur radicals may benefit from re-evaluation in models.

**Scheme 1:**
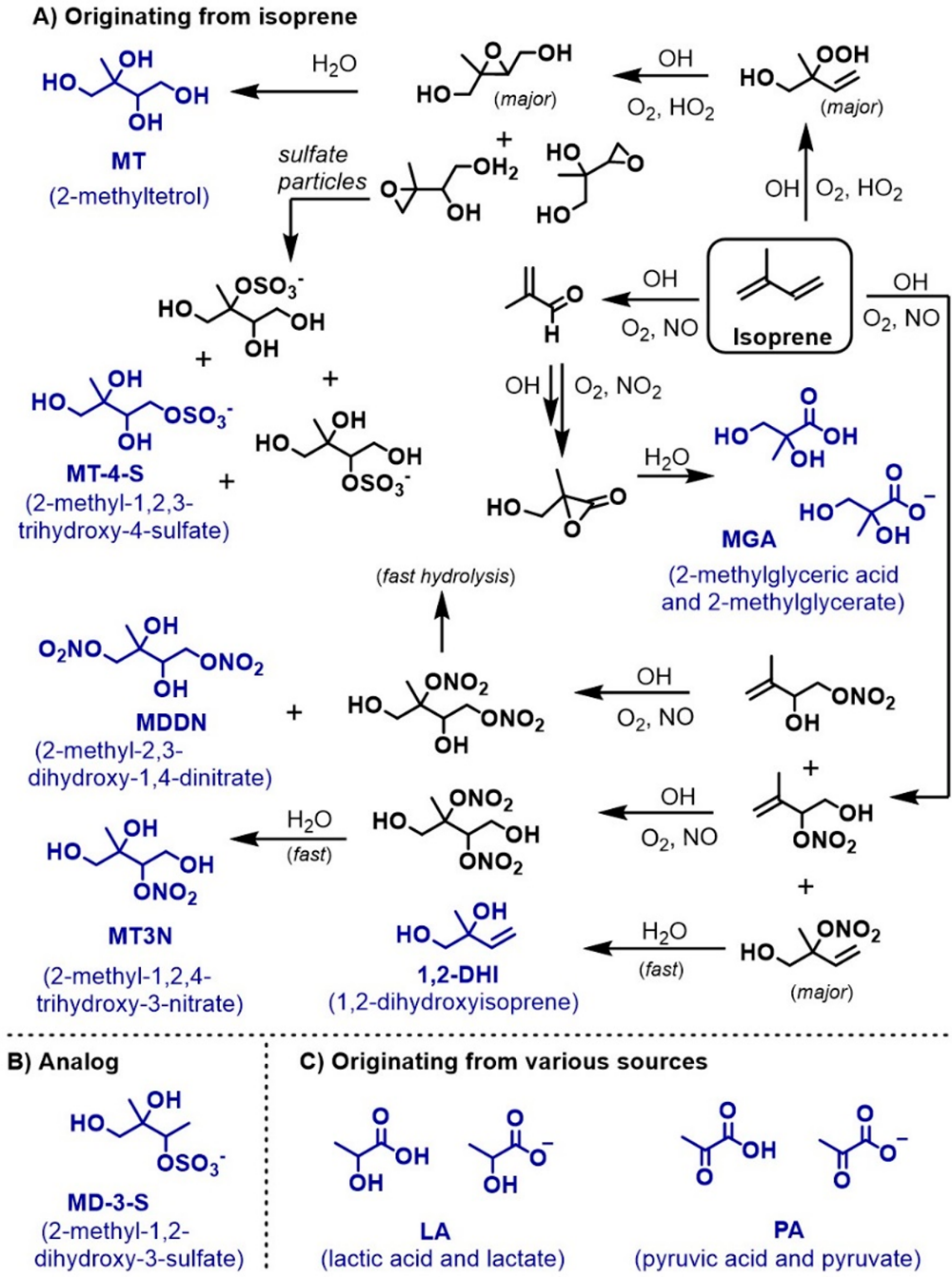
(A) Compounds Originating from Isoprene, (B) a Structural Analog to Isoprene’s Organosulfate, and (C) Compounds Originating from Various and Often Multiple Sources in the Atmosphere Compounds under study are shown in blue. The isoprene scheme is derived from Wennberg et al.^1^

With this in mind, chemical transport models need second-order SO_4_^•–^ rate coefficients (*k*_SO4_) for water-soluble organic compounds to better represent condensed-phase chemistry. However, *k*_SO4_ rate coefficients for complex compounds present in biogenic organic aerosols are largely unknown.^5,24−26^ As these compounds may contain a diverse array of functional groups, effort may be required to synthesize and purify them, and a multitude of instrumental methods are required for their analysis. Previously, we reported the second-order rate coefficients for aqueous hydroxyl radical (OH) oxidation (*k*_OH_) for several oxidation products of isoprene obtained through chemical synthesis, which were measured using competitive rate experiments with erythritol.^27^ We follow up here with their *k*_SO4_ rates and add some additional organic acids to the study. Detailed justifications for the study of the isoprene-related species were presented in-depth previously, along with the synthetic and analytical method development. Briefly, the isoprene-derived compounds under study are either formed from the gas-phase oxidation of isoprene^1^ and partitioned to the condensed phase due to their low vapor pressure or formed after a multiphase reaction (Scheme 1A). these compounds include products from the low-NO channel, including 2-methyltetrol (MT) and 2-methyl-1,2,3-trihydroxy-4-sulfate (MT-4-S),^28−32^ and from the high-NO channel, including 2-methyl-2,3-diol-1,4-dinitrate (MD-1,4-DN), 2-methyl-1,2,4-triol-3-nitrate (MT-3-N),^33^ 1,2-dihydroxyisoprene (1,2-DHI, referred to as MDE in our previous work)^39−41^ and 2-methylglyceric acid (MGA).^34,35^ A structural analog of isoprene’s organosulfate, 2-methyl-1,2-dihydroxy-3-sulfate (MD-3-S), was obtained as a side product in the chemical synthesis of MT-4-S and studied here to gain insight into the structural reactivity. In this work, we also examine some small organic acids such as lactic acid (LA) and pyruvic acid (PA) because they are ubiquitous in the atmospheric condensed phase.^36^

## Methods

### Materials and Synthesis

Erythritol (99%), xylitol (≥99%), potassium persulfate (K_2_S_2_O_8_, 99%), acetonitrile (99%), and pyruvic acid (≥99%) were obtained from Sigma-Aldrich. α-Methylglyceric acid (M311505, 98%) was purchased from Toronto Research Chemical s (TRC), Inc. Formic acid (≥99%) was purchased from Thermofisher Scientific. Lactic acid (90% in water) was purchased from Acros Chemicals. All reagents were used without further purification. Compounds were dissolved and diluted using ultrapure H_2_O from a Milli-Q purification system (Millipore Sigma, 18 MΩ cm^–1^). 2-Methyltetrol (MT), 1,2-dihyroxyisoprene (1,2-DHI), 2-methyl-1,2,3-trihydroxy-4-sulfate (MT-4-S), and 2-methyl-1,2-dihydroxy-3-sulfate anion (MD-3-S) were synthesized and purified according to the literature;^37^ their nuclear magnetic resonance (NMR) spectra matched previously reported data. The organosulfates were purified for use over an ion-exchange column (Dowex 50WX4, 50–100 mesh). 2-Methyl-2,3-dihydroxy-1,4-dinitrate (MD-1,4-DN) and 2-methyl-1,2,4-trihydroxy-3-nitrate (MT-3-N) were synthesized previously by our group.^27^ All synthesized chemicals used in this work had purities >95%, as determined by ^1^H NMR. Due to the relative rate technique and the accurate-mass or structure-specific analytical measurements, organic impurities are not expected to alter the reaction kinetic ratios.

### Experiments

Photochemical reactions of organic compounds of interest with sulfate radicals were examined in quartz reaction vessels using the UV irradiation of potassium persulfate (K_2_S_2_O_8_) as a sulfate radical precursor. Similarly to other studies, the stable reactant was used in large excess relative to SO_4_^•–^ in order to ensure pseudo steady-state kinetics.^38^
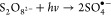
i
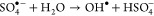
ii
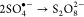
iii

A relative rate technique^39^ was used, where the second-order rate coefficients for the compound of interest with respect to oxidation by the SO_4_^•–^ radical were determined against a reference compound with a known rate coefficient in the same reaction. The reference compound selected for study was erythritol (C_4_H_10_O_4_), which has a rate constant of 4.56 × 10^7^ M^–1^ s^–1^ with the sulfate radical when averaged over the experimental temperature range.^24,40^
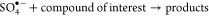
1
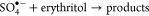
2

Under the conditions of our study, *J*_i_ is ∼5.5 × 10^–5^ s^–1^. Using known rate coefficients for the above reactions (e.g., *k*_ii_ ∼ 400 s^–1^)^41^ and those of erythritol with OH and SO_4_^•–^,^4^ it was estimated that >99% of the reaction proceeded via SO_4_^•–^ compared to OH (Table S1).

Rate coefficients for the reactions were obtained as follows:
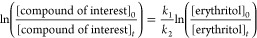
where [compound of interest]_0_ and [erythritol]_0_ are the respective chromatographic or spectroscopic peak areas for those species at time zero, [compound of interest]_*t*_ and [erythritol]_*t*_ are the peak areas at time *t*, and *k*_1_ and *k*_2_ are the bimolecular rate coefficients for reactions 1 and 2, respectively. Plotting ln([erythritol]_0_/[erythritol]_*t*_) and ln([compound of interest]_0_/[compound of interest]_t_) generates a linear plot in the positive quadrant, where *k*_1_/*k*_2_ is the slope of the line.

**Figure 1:**
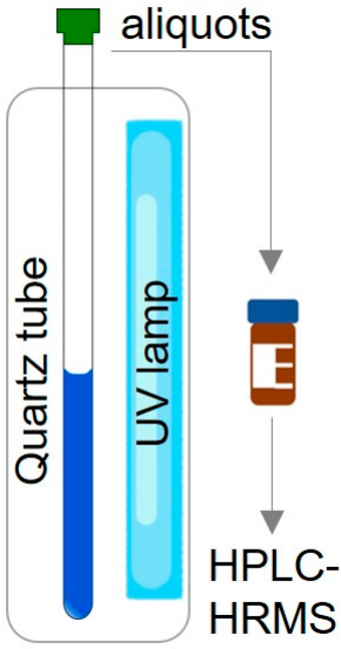
Experimental set-up. A quartz reaction tube within a glass compartment is exposed to a UV lamp. Aliquots of the sample at different time points are diluted and analyzed via HPLC-HRMS.

**Figure 2:**
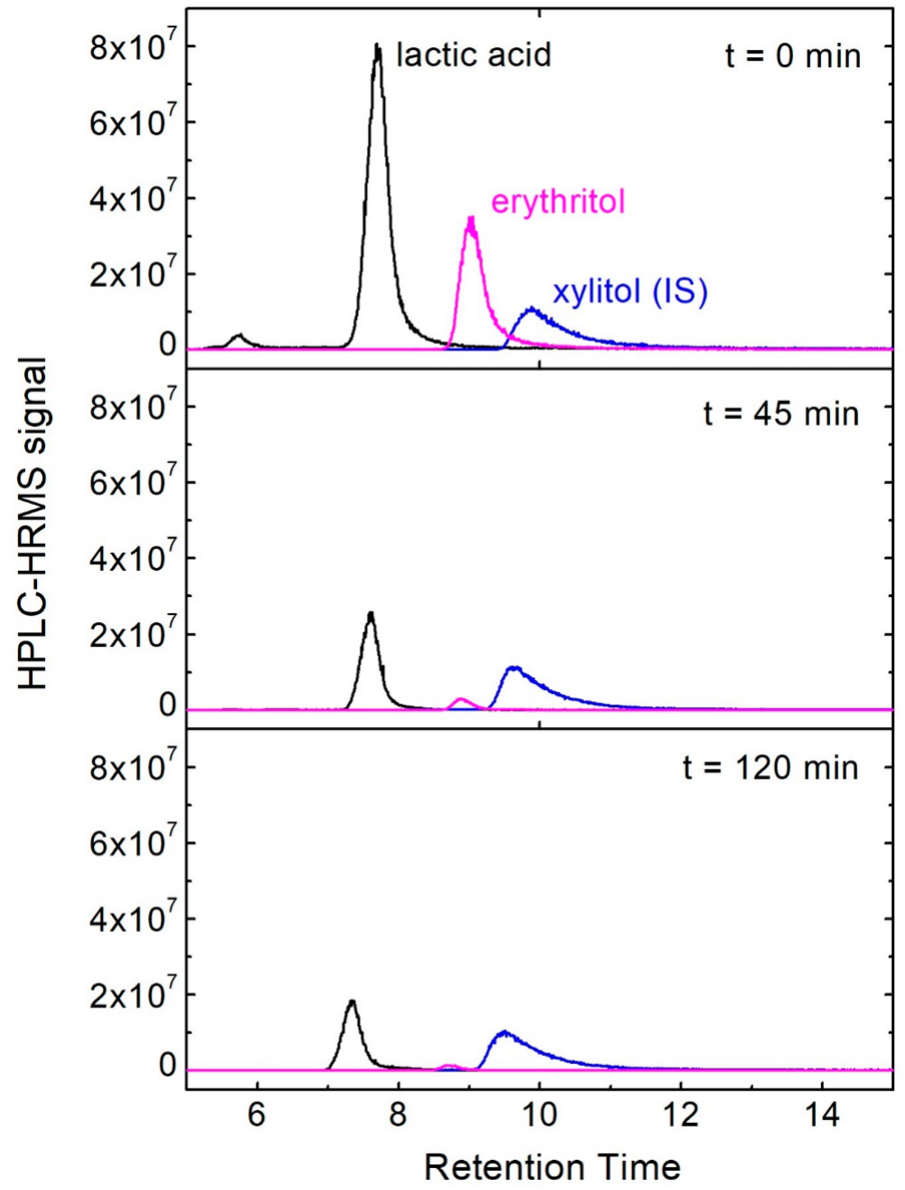
Representative HPLC-HRMS data for a kinetics experiment using lactic acid against erythritol at pH 2. Xylitol was added as an internal standard (IS). Data are normalized to the IS peak area.

Solutions of 5 mM erythritol, 15–20 mM K_2_S_2_O_8_, and 5 mM compound of interest were prepared in Milli-Q water for experiments. The unadjusted pH values for MGA, PA, and LA were 3, 5, and 2, respectively. To analyze 2-methylglycerate and lactate (pH 5), NaOH was added dropwise to the mixed solution until pH 5 was reached. MGA and pyruvic acid were analyzed at pH 2 by adding H_2_SO_4_ dropwise to the mixed solution until pH 2 was reached. All other determinations were performed at pH 5–7. A quartz reaction vessel was filled with 10–15 mL of the sample mixture, placed in an enclosed compartment with a 254 nm UV lamp, and irradiated over the course of two hours (See Figure 1 for the experimental setup). Using 400 μL H_2_O spiked with xylitol (0.762 mM) as an internal standard, 100 μL aliquots of the reaction solution were analyzed at variable time points after dilution. Representative data are shown in Figure 2. All experiments were performed in triplicate, and uncertainty bounds represent one standard deviation.

**Figure 3:**
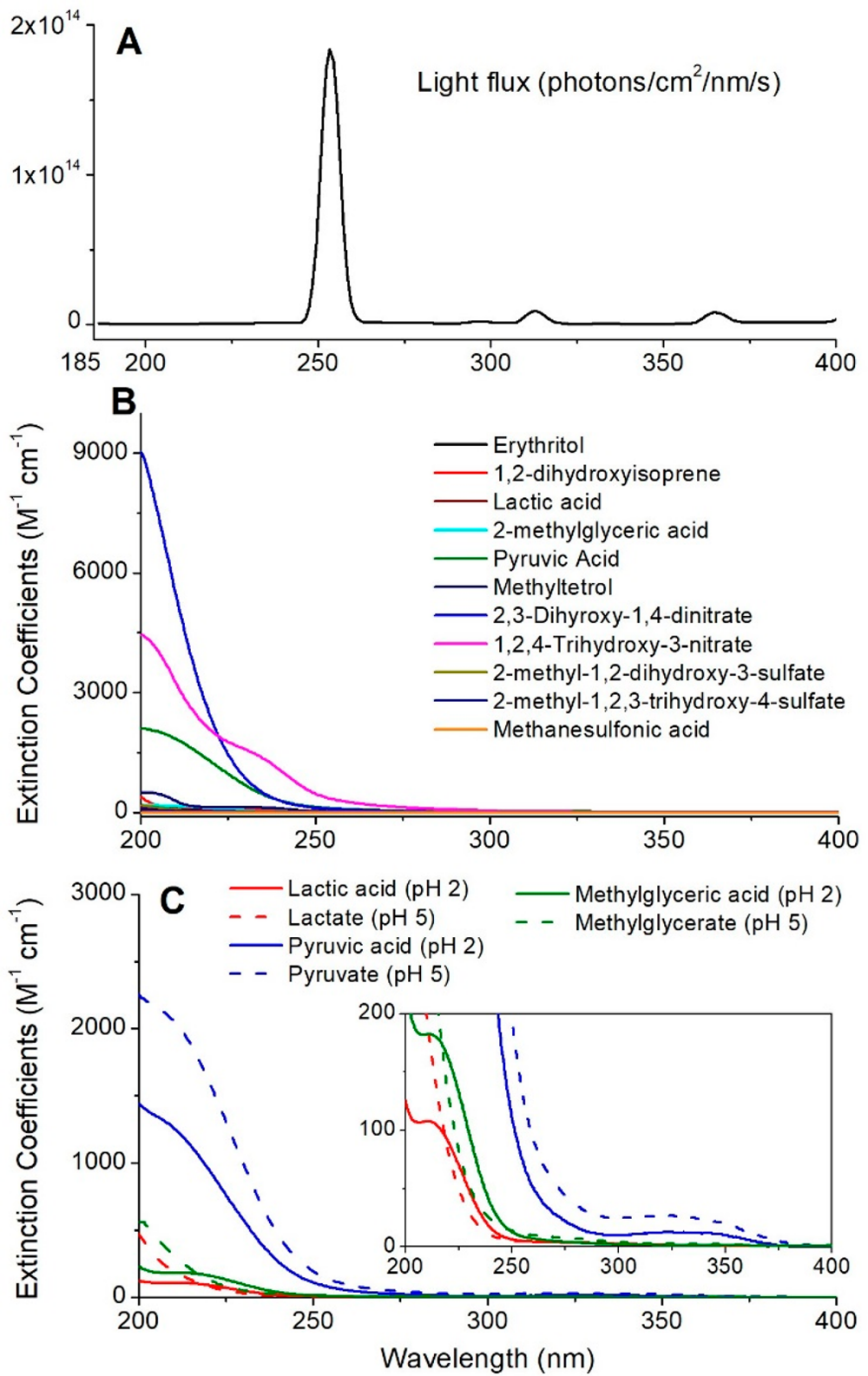
(A) Wavelength-dependent photon flux from the UV lamp, which was calibrated using chemical actinometry. Optical extinction coefficients (M^–1^ cm^–1^) for (B) all compounds under study in pure water at unadjusted pH and (C) for the carboxylic acids at two pH values. The insert in panel C shows a magnified view of the absorbance features of organic acids in the 200–400 nm region.

Direct photolysis controls for compounds with chromophoric functional groups were performed identically to the experiment except without the erythritol reference and without K_2_S_2_O_8_. The spectral output from the lamp was measured using a portable spectrophotometer (Tidas series, WPI Inc.), and the photo flux (Figure 3A) was calibrated using the neutral aqueous photolysis of uridine as a chemical actinometer according to protocol L09 from IUPAC.^42^ Erythritol and other compounds without chromophores are assumed to undergo negligible photolysis during the experiment. A first-order loss rate (*J*) was extracted from the control experiments for the compounds of interest, and a correction to the raw data was performed where the *k*_1_/*k*_2_ ratio was obtained by plotting ln([erythritol]_0_/[erythritol]_*t*_) against ln([compound of interest]_0_/[compound of interest]_*t*_ – *J* × *t*). For experiments with photolysis controls, the uncertainties in the *J* determinations (e.g., errors in slopes) were propagated together with the standard deviations from the data.

In order to understand the temperature fluctuations during an experiment, a control experiment with Milli-Q water was performed with continuous irradiation while the temperature was monitored with a thermocouple (Figure S1). The temperature of the solution was recorded until temperature stabilization occurred. Thus, data are reported in the range of 298–322 K. The studied compounds are stable at these temperatures. Although the kinetic data are referenced and SO_4_^•–^ reaction rates with similar compounds are weakly dependent on temperature,^26,40^ it is not clear whether the temperature dependence of the rate coefficient for the reference compound is identical to that of the compound of interest. Partitioning to the gas phase may increase at higher temperatures in the capped quartz tube; however, all studied compounds have large Henry’s Law coefficients that favor the aqueous phase by many orders of magnitude. 1,2-DHI is likely the most volatile compound in the study; its Henry’s law coefficient may be estimated based on 1,2-pentanediol (*K*_H_ = 1.4 × 10^5^ M atm^–1^ or 3.4 × 10^6^ mole ratio of aqueous to gas).^43^ The temperature uncertainty can be considered a limitation of this work.

### Analytical Measurements

All analytes, with the exception of 1,2-DHI, were analyzed using high-performance liquid chromatography (HPLC) coupled to high-resolution mass spectrometry (HRMS). The HPLC-HRMS analyses were performed using an Agilent 1100 HPLC coupled to a linear-trap-quadrupole Orbitrap (LTQ-Orbitrap) mass spectrometer (Thermo Corp., Waltham MA) operated at a mass resolving power of 60 000 m/Δm at *m*/*z* 400. Xcalibur 2.0 software was used to analyze the mass spectra. The separation of MT, MD-1,4-DN, MT-3-N, erythritol, and xylitol was performed using a Shodex Asahipak NH2P-40 2D column (2 × 150 mm, 4 μm, 100 Å) using a method recently reported elsewhere.^27^ An Agilent Poroshell InfinityLab EC-C18 (2.1 × 100 mm, 2.7 μm, 120 Å) column was used to separate MD-3-S and MT-4-S using a previously reported method.^27^ To analyze pyruvic acid, lactic acid, and MGA, including erythritol and xylitol, a Shodex HILICpak VG-50 2D column (2 × 150 mm,5 μm, 100 Å) was used. The mobile phases were MeCN (A) and 0.5% NH_3_ (B), and the injection volume was 1 μL. To separate MGA from erythritol and xylitol, the mobile phase was held at 30% B for 2 min, increased to 90% B for 10 min, held at 90% for 3 min, decreased to 30% for 5 min, and held at 30% for another 5 min. The flow rate was 0.3 mL min^–1^, and the total run time was 25 min. To separate pyruvic acid and lactic acid from erythritol and xylitol, the mobile phase was held at 30% B for 4 min, increased to 90% for 6 min, held at 90% for 1 min, decreased to 30% for 4 min, and held at 30% for 2 min. The flow rate was 0.1 mL min^–1^, and the total run time was 17 min. A representative HPLC-HRMS data set for lactic acid is shown in Figure 2.

For the rate determinations of 1,2-DHI, proton nuclear magnetic resonance (^1^H NMR) spectroscopy was used to quantify the decay of erythritol and 1,2-DHI due to the distinct proton environments of the alkene using a method similar to that we reported previously.^27^ 1,2-DHI is poorly ionizable in the HPLC-HRMS method. Due to the lower sensitivity of the NMR analysis, 100 mM concentrations of both reactants were mixed with 300 mM K_2_S_2_O_8_ in order to increase the analytical signal. Reactions for NMR analyses were performed on a 400 MHz Bruker instrument (400 MHz Bruker Avance III HD Nanobay Spectrometer) using an autosampler and analyzed using TOPSPIN. The solution was irradiated over the course of 1.5 h and taken to the instrument without any workup at several time points.

A Shimadzu UV-1800 dual-beam spectrophotometer was used to measure the UV–visible extinction coefficients of organic reactants as referenced with pure water, primarily to assess which reactant required a direct photolysis correction. A 1 cm quartz cuvette was used for the absorbance measurements for 0.1–1 mM samples, which were diluted in order to maintain Beer’s Law linearity when required. Extinction coefficients were then calculated based on the measured absorbances, the path length, and the known concentrations of the solution.

## Results and Discussion

### Direct Photolysis

It was found that pyruvic acid (PA, pH 2 and pH 5), 1,2-DHI, MD-1,4-DN, and MT-3-N had non-negligible absorption cross sections (>20 M^–1^ cm^–1^) at 254 nm (Figure 3) that required a direct photolysis correction. A magnified view of Figure 3B is shown in Figure S2. These compounds have chromophores such as carbonyl, alkenyl, and nitrato groups, so their absorbance in the 200–400 nm range is expected. Pyruvic acid has a carbonyl in the β-position relative to the acid, which increases its chromophoric properties compared to those of hydroxyacids such as lactic acid. Photolysis controls performed for these compounds (Figure S3) yielded first-order loss rates of approximately 5–40 × 10^–5^ s^–1^, which were used to correct the rate ratios against erythritol. Quantum yields under our lamp could be estimated to be in the range of ∼0.1–0.2 for the organonitrates and pyruvate (pH 5) but were larger than unity for pyruvic acid (PA) and 1,2-DHI (Table S2). Eugene et al.^44^ reported that the photolytic loss of PA in water at pH 1 proceeds with a quantum yield of ∼2, in good agreement with our estimate (2.4). Effectively, each PA* molecule that is excited per absorbed photon consumes another molecule of PA in a highly efficient bimolecular process. Although the chromophore-loss quantum yield of pyruvate in water has not been reported, to our knowledge, the decarboxylation quantum yield for pyruvate is at least twenty times smaller than that for the acid,^45^ in qualitative agreement with the much smaller quantum yield we extracted for pyruvate loss (0.19). Aqueous photolysis data for other compounds studied in this work were not available in the literature for further comparisons.

### Kinetics Experiments

**Figure 4:**
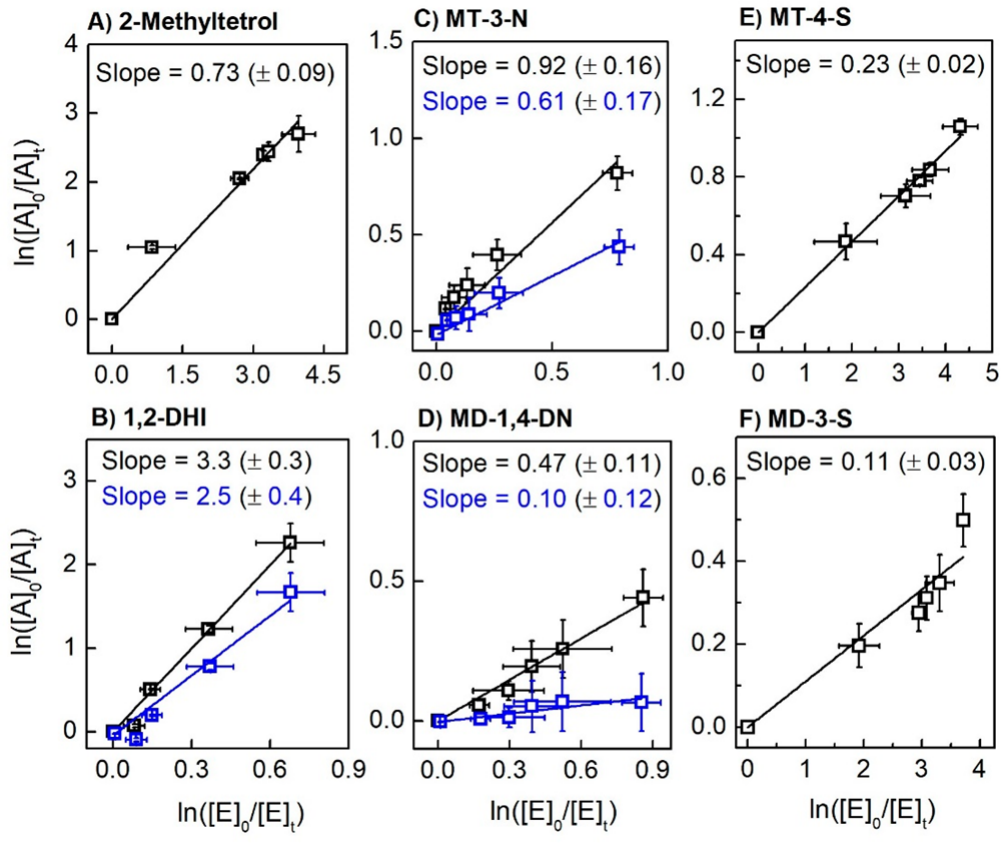
Relative rate determinations for the isoprene-derived polyols, organonitrates, and organosulfates (“A”) against reference erythritol (“E”). Data shown in blue are corrected for direct photolysis. (A) 2-Methyltetrol, (B) 1,2-dihydroxyisoprene (1,2-DHI), (C) 2-methyl-1,2,4-trihydroxy-3-nitrate (MT-3-N), (D) 2-methyl-2,3-dihydroxy-1,4-dinitrate (MD-1,4-DN), (E) 2-methyl-1,2,3-trihydroxy-4-sulfate (MT-4-S), and (F) 2-methyl-1,2-dihydroxy-3-sulfate (MD-3-S). A linear least-squares fit assumes a *y*-intercept of zero. Chemical structures are shown in Scheme 1.

**Figure 5:**
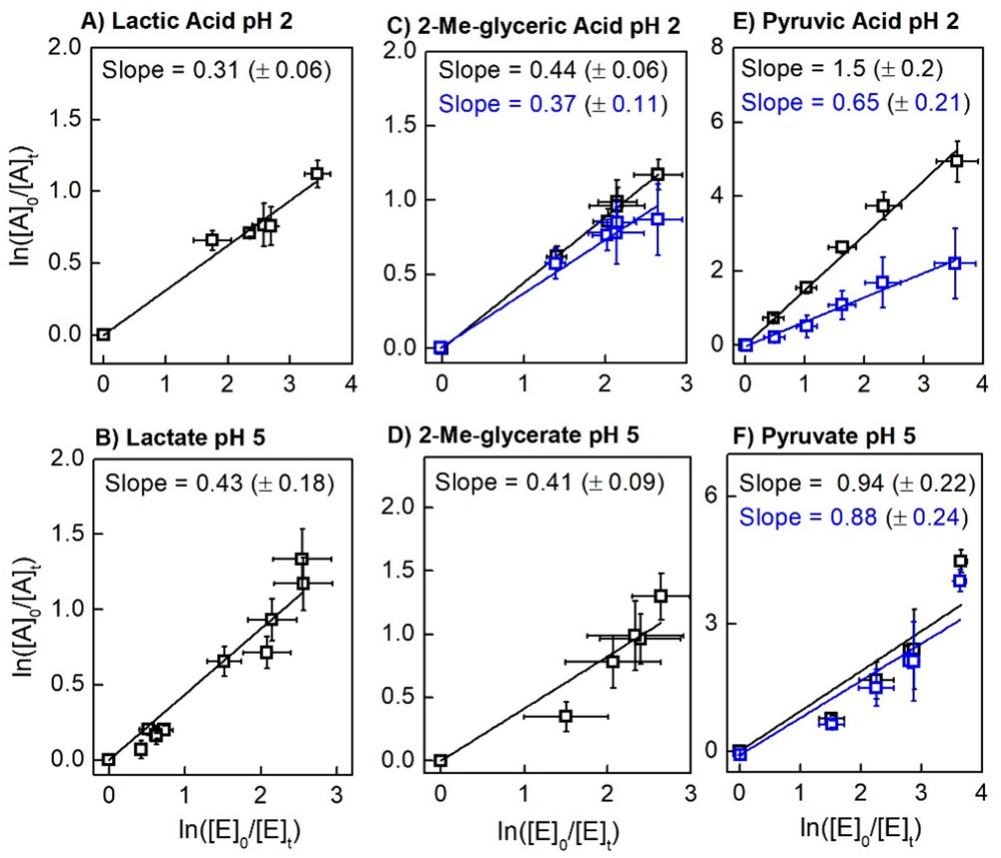
Relative rate determinations for carboxylic acid compounds of interest and their carboxylates (“A”) against reference compound erythritol (“E”). (A) Lactic acid, (B) lactate, (C) 2-methylglyceric acid, (D) 2-methylglycerate, (E) pyruvic acid, and (F) pyruvate. Data shown in blue are corrected for direct photolysis. A linear least-squares fit assumes a *y*-intercept of zero. Chemical structures are shown in Scheme 1.

From the decay of erythritol, the steady-state concentrations of SO_4_^•–^ used for experiments were determined in the range of 0.1–2 × 10^–11^ M. Ratios of *k*_1_/*k*_2_ for the different compounds of interest, derived from triplicate trials, are shown in Figures 4 and 5. Data points were fit linearly, with the slope being “*k*_1_/*k*_2_”. Direct photolysis corrections are shown in blue where appropriate. Table 1 shows the *k*_SO4_ values for the compounds under study after the application of the rate coefficient of erythritol to the respective rate ratios. Control experiments did not show appreciable reactions between organics and the K_2_S_2_O_8_ reagent in the dark at the concentrations and time scales relevant to this work (Figure S4).

**Table 1:** Measured Second-Order *k*_SO4_ Rate Coefficients in Water in the Range of 298–322 K^a^

compound	abbreviation	*k*_SO4_ (M^–1^ s^–1^)^b^
2-methyltetrol	MT	3.3 (±0.4) × 10^7^
2-methyl-2,3-dihydroxy-1,4-dinitrate	MD-1,4-DN	4.6 (±5.0) × 10^6^
2-methyl-1,2,4-trihydroxy-3-nitrate	MT-3-N	2.8 (±0.7) × 10^7^
1,2-dihydroxyisoprene	1,2-DHI	1.1 (±0.2) × 10^8^
2-methyl-1,2,3-trihydroxy-4-sulfate	MT-4-S	1.1 (±0.8) × 10^7^
2-methyl-1,2-dihydroxy-3-sulfate	MD-3-S	5.0 (±1.3) × 10^6^
2-methylglyceric acid	MGA (pH 2)	1.7 (±0.5) × 10^7^
	MGA (pH 5)	1.9 (±0.4) × 10^7^
Lactic acid	LA (pH 2)	1.4 (±0.3) × 10^7^
	LA (pH 5)	2.0 (±0.8) × 10^7^
Pyruvic acid	PA (pH 2)	3.0 (±0.9) × 10^7^
	PA (pH 5)	4.0 (±1.4) × 10^7^

a Direct photolysis corrections have been applied, where appropriate. Chemical structures are shown in Scheme 1.

b Measured against reference compound erythritol (4.56 × 10^7^ M^–1^ s^–1^)

**Scheme 2:**
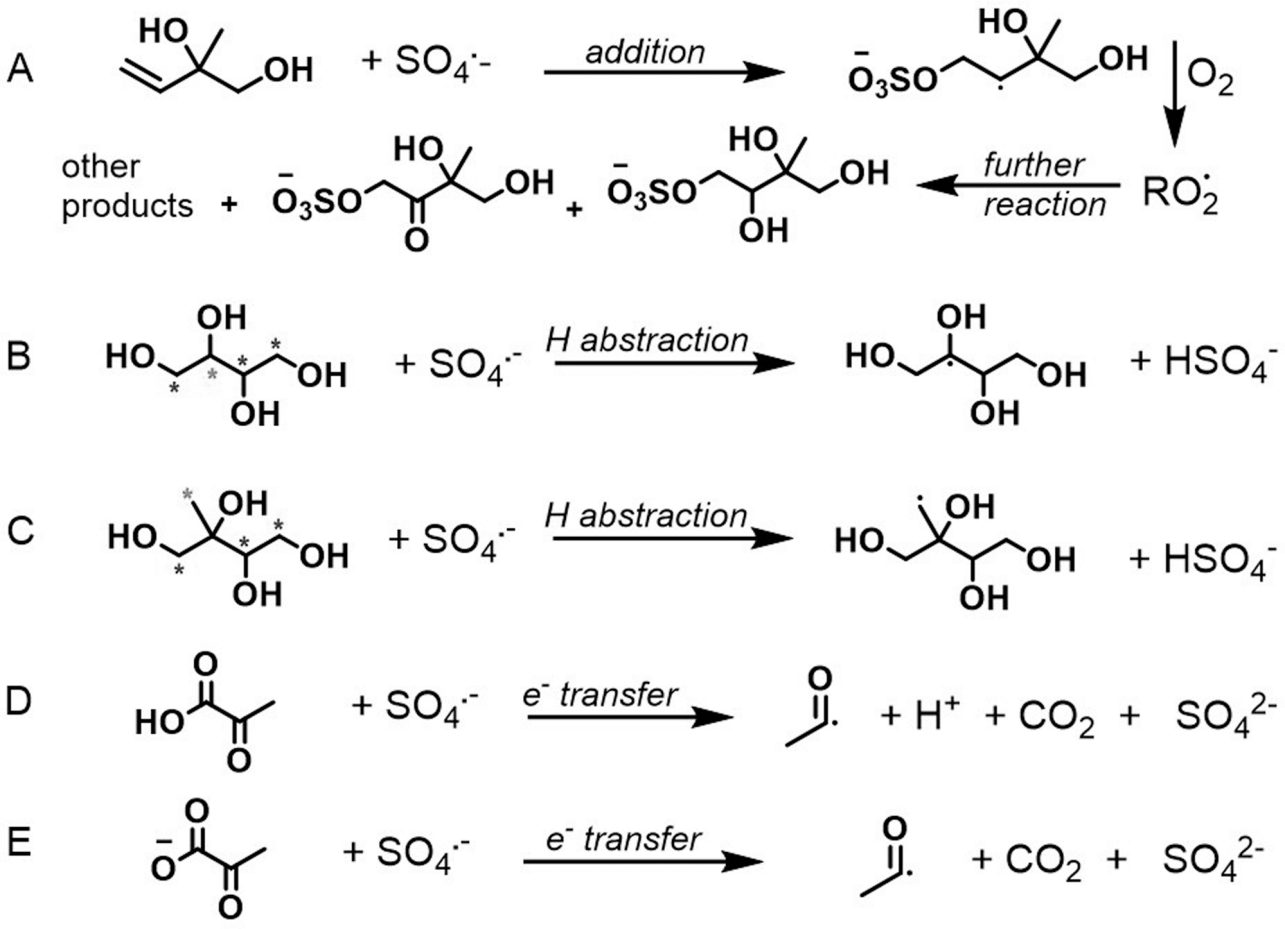
Representative Examples of Reactions of the Sulfate Radical (A) The reaction with 1,2-DHI through addition to the double bond, which forms an alkylperoxy radical (RO_2_) in the presence of oxygen and eventually stable organosulfate products through RO_2_ + RO_2_ chemistry and other reactions. (B) H-atom abstraction from erythritol versus (C) 2-methyltetrol, where the blue asterisks denote identical abstraction sites and the magenta asterisks denote different abstraction sites between the two compounds. (D) The direct electron transfer reaction of carboxylic acids and (E) their carboxylates, which results in a decarboxylation reaction that propagates carbon-centered radicals. The reactions shown here do not represent all reactive pathways that may occur for each compound of interest.

Starting with the isoprene-derived polyols, organonitrates, and organosulfates (Figure 4), we see the largest rate coefficient in the reaction of 1,2-DHI versus erythritol. The unsaturation in 1,2-DHI enables an addition mechanism for SO_4_^•–^ that is unavailable for the other compounds under study (Scheme 2a); thus, the reaction of SO_4_^•–^ with 1,2-DHI is expected to contribute to the formation of organosulfates.^8,9^ The aqueous SO_4_^•–^ rate coefficient of 1,2-DHI for this work (1.14 × 10^8^ M^–1^ s^–1^) is comparable to SO_4_^•–^ rate coefficients that have been reported for other atmospherically relevant alkenes, such as methacrolein and methylvinylketone (9.9 × 10^7^ and 1.1 × 10^8^ M^–1^ s^–1^, respectively).^25^ The reaction of MT is ∼30% slower than that of erythritol, which may be rationalized on the basis of their structural differences. MT has the same basic structure as erythritol but has an additional methyl group at the second carbon; thus, MT has a less-reactive primary-carbon abstraction site (R-(HO)(C**H**_**3**_)C-R) compared to the secondary-carbon abstraction site (R-(HO)**H**C-R) of erythritol (Scheme 2b and c). For the organic dihydroxy dinitrates of isoprene,^33^ we expect the major isomer MD-2,3-DN to be formed from gas-phase oxidation. However, once formed and partitioned to the condensed phase, its aqueous oxidation will not be competitive with the fast hydrolysis fate.^46,47^ The hydrolysis product formed from MD-2,3-DN, namely, 2-methyl-1,2,4-triol-3-nitrate (MT-3-N), was studied here due to its higher relevance for the aqueous reaction. The mononitrate MT-3-N participated in direct photolysis as well as the SO_4_^•–^ reaction, and the corrected rate coefficient (∼3 × 10^7^ M^–1^ s^–1^) was similar to that of MT within uncertainty. This could potentially suggest that substitution of an OH group with a ONO_2_ group at a secondary carbon does not significantly change the SO_4_^•–^ reactivity. As opposed to the major dinitrate MD-2,3-DN, the minor dinitrate isomers (nitrates at the 1,4- and 1,3-positions) are expected to build up in concentration in the condensed phase given their slow hydrolysis fates.^48^ Thus, the aqueous oxidation of the minor dinitrate isomers such as MD-1,4-DN would be a competitive fate. However, we found that MD-1,4-DN was relatively slow with respect to its reaction with SO_4_^•–^ (∼5 × 10^6^ M^–1^ s^–1^). Its reaction with OH radicals is also relatively slow.^27^ It is not clear at this time whether this finding is relevant to nitrates in the primary position or whether substitution with two nitrate groups has outsized effects compared to one. Relative to its rate coefficient with aqueous OH (∼2 × 10^8^ M^–1^ s^–1^),^27^ the SO_4_^•–^ reaction of MD-1,4-DN may not be competitive unless the particle phase supplies a large reservoir of sulfate radicals.

We studied the organosulfates as their sodium salts, as the p*K*_a_ values of these species are expected to be negative.^49^ Both the primary organosulfate, MT-4-S (∼1 × 10^7^ M^–1^ s^–1^), and the secondary organosulfate, MD-3-S (∼5 × 10^6^ M^–1^ s^–1^), had low reactivities with SO_4_^•–^; however, the third OH group in MD-3-S is absent, which does not allow for an exact structural comparison. This is drastically different from the OH rates, where MT-4-S has a *k*_OH_ rate only 20% slower than that for erythritol. MT-4-S has a faster *k*_SO4_ than MD-3-S (about two times faster), a trend similar to what we observe with the OH radical. Within organic aerosols of isoprene, the sulfate in the tertiary position is expected to be the major isomer, either from the reaction of the sulfate radical with isoprene^50^ or from the ring opening of the isoprene epoxydiols,^51^ but the secondary species is also present. The equivalent primary OS has not been observed, although it was studied here due to the enhanced feasibility of its organic synthesis. Unlike the tertiary nitrates of isoprene, the tertiary sulfate is fairly stable with respect to hydrolysis,^52^ so oxidation is a competitive fate. It is presumed that the SO_4_^•–^ rate of the tertiary sulfate will be within a factor of two or three of the primary sulfate, assuming general substitution trends from OH oxidation can be applied here.^53−58^ With this assumption, we can estimate a lower limit of 3.5 × 10^6^ M^–1^ s^–1^ for the tertiary species.

A number of organic acids that have been observed in atmospheric aerosols^59^ were investigated in this study. As these acids exist in the environment as either their neutral or anionic forms depending on the pH, two atmospherically relevant pH values were studied: pH 2 for relevance to sulfate-based aerosols and pH 5 for relevance to cloud droplets. In general, we found the *k*_SO4_ coefficients of the organic acids under study to be slightly lower at pH 2 compared to pH 5 (Figure 5), although in some cases it was challenging to reach this conclusion within uncertainty. This general trend was also observed for other organic acids, with differences between the reaction of the carboxylate and the acid most severe for formic acid (∼2 orders of magnitude) and less so for larger acids and diacids (within 10% for malic acid).^60^ SO_4_^•–^ radicals efficiently decarboxylate carboxylic acids through electron transfer (Scheme 2d and e),^61^ and the carboxylate form facilitates this reaction for some compounds.^62^ For lactate at pH 5, our *k*_SO4_ coefficient (∼2 × 10^7^ M^–1^ s^–1^) in the range of 293–322 K is in reasonable agreement with a previously determined value (∼1.6 × 10^7^ M^–1^ s^–1^) at pH 9, which was controlled at 298 K.^60^ Our value for lactic acid at pH 2 (∼1.4 × 10^7^ M^–1^ s^–1^) is also within error of the value determined previously (∼1.0 × 10^7^ M^–1^ s^–1^ at pH 1); discrepancies are likely due to the temperature difference between our study and the cited study. Methylglycerate at pH 5 was found to have a *k*_SO4_ similar to that of lactate. Both methylglycerate and lactate have one −CH_2_– carbon and one −CH_3_ carbon, so perhaps their similar rate coefficients can be rationalized through structural similarities. Methylglyceric acid (MGA) at pH 2 was originally determined to have a slightly higher rate coefficient than methylglycerate; however, there is possibly a photolysis correction that should be applied. Although MGA did not have a high extinction coefficient at 254 nm (∼10 M^–1^ cm^–1^), the possibility for a quantum yield higher than unity could make direct photolysis a concern. We did not have additional MGA samples with which to study the direct photolysis. We applied the same chromophore loss quantum yield for pyruvic acid to estimate a maximum correction due to direct photolysis and found that up to a 15% reduction in *k*_SO4_ could be expected if MGA behaved like PA with respect to direct photolysis. We do not expect MGA to be quite as photolytically labile as PA as it is missing the carbonyl chromophore of PA. Within error, we conclude that MGA has a rate coefficient similar to methylglycerate. Otto, Schaefer, and Herrmann^26^ found a weak pH dependence for the *k*_SO4_ of terpene-derived organic acids, an observation that also applies to this data set.

The SO_4_^•–^ reaction of pyruvate was found to be twice as fast as those of lactate and methylglycerate. The direct photolysis of pyruvate was slow; thus, the correction is fairly minor at pH 5. At pH 2, pyruvic acid (PA) has the fastest direct photolysis rate (Figure S3), which led to a greater than 50% correction to its observed rate of decay. PA appears slower than pyruvate in its SO_4_^•–^ reaction after the correction, although this is not clear within uncertainty. It is safe to say, however, that PA reacts faster with SO_4_^•–^ than the other studied carboxylic acids. The data here appear to be in good agreement with those of Otto, Schaefer, and Herrmann,^26^ who found the *k*_SO4_ of *cis*-pinonic acid (a keto acid) to be ∼3 × 10^7^ M^–1^ s^–1^ and that of camphoric acid (diacid without the carbonyl group) to be roughly half this value. It is possible that a carbonyl group in the structure of an organic acid increases the rate of reaction with SO_4_**^•–^** due to the production of an acyl radical (Scheme 2 d and e) compared to an alkyl radical, but this remains to be further explored.

## Conclusions

We reported second-order SO_4_^•–^ rate constants for a number of atmospherically relevant compounds (Table 1). Within the studied compounds, we found the alkene 1,2-DHI reacted the fastest with SO_4_^•–^ and the dihydroxy (primary) dinitrate MD-1,4-DN reacted the slowest, which is similar to their relative reactivities with OH radicals.^27^ There are also different trends that may highlight the greater selectivity of SO_4_^•–^ reactions; for example, MT-4-S reacted with SO_4_^•–^ faster than MT when that trend was reversed with OH. The C_3_–C_5_ acids under study did not have significantly different reactivities with SO_4_**^•–^** compared to their carboxylates within the uncertainties of the analyses. Of particular interest may be the reaction of 1,2-DHI with SO_4_^•–^, which is able to form surface-active organosulfates,^8−10,50^ or the potential reaction of MT-4-S with SO_4_^•–^ to propagate sulfur radicals.^21^ For other compounds, the *k*_SO4_ rates determined here may help improve the understanding of aerosol mass loss, which may be underpredicted in current models.^63^ However, even with a better understanding of *k*_SO4_ and *k*_OH_ rate coefficients, a comparison of SO_4_^•–^ versus OH sinks for various organics may still be qualitative at this time. Oxidant concentrations in deliquesced particles and clouds are major sources of uncertainty in multiphase modeling. Models predict a vast range of steady-state OH concentrations in the atmospheric aqueous phase^64^ and often overestimate [OH]_aq_ by orders of magnitude compared to measured values that have been reported within quite a narrow range ((0.1–0.6) × 10^–15^ M for aerosols).^65−67^ While revisions of state-of-the-art model mechanisms such as CAPRAM significantly reduce the predicted OH concentrations compared to previous versions,^64^ the SO_4_^•–^ concentrations in models have not yet been revised, potentially due to the lower amount of available data. Aerosol water SO_4_^•–^ concentrations in urban-influenced environments are predicted to be roughly 1 × 10^–14^ M in the most recent version of CAPRAM. However, there are no measured SO_4_^•–^ concentrations in deliquesced aerosols or cloud or fogwater with which to compare these values. If we keep [SO_4_^•–^]_aq_ at 1 × 10^–14^ M and allow the full range of measured [OH]_aq_ to compete, the SO_4_^•–^ reaction would span from 10–70% of the summed reactivity of the organic using kinetic data reported by our group (assuming 2-methyltetrol and 1,2-DHI as the organics). We ignore the reactivity with NO_3_ radical for now, as its rate coefficients with the compounds under study have yet not been determined. Thus, there may be situations in mixed urban–biogenic environments (such as regions of the Southeast US) for SO_4_^•–^ to be a competitive fate of some organics in aerosols; however, better constraints on aqueous oxidant sources are likely needed in order to improve the understanding of the reactive fates of aerosol-phase compounds.

## Supporting Information Available

Kinetic competition calculation between SO_4_^•–^ and OH, direct photolysis parameters, photolysis correction calculations, temperature of the reaction medium, magnified view of extinction coefficients, plot of direct photolysis control data, and plot of dark control data (PDF)

## Notes

Data are publicly available in the Dryad Repository (https://datadryad.org/) under the following DOIs: optical extinction coefficients (10.25338/B83M0M) and relative rate kinetic data (10.25338/B8V34S).
