# Supplementary material for: Second-Order Kinetic Rate Coefficients for the Aqueous-Phase Sulfate Radical (SO4•–) Oxidation of Some Atmospherically Relevant Organic Compounds
Source: J Phys Chem A. 2022 Sep 7;126(37):6517–25. doi: 10.1021/acs.jpca.2c04964 (PMC9511566; doi:10.1021/acs.jpca.2c04964)
Supplement: Supplementary file 1 — jp2c04964_si_001.pdf [file jp2c04964_si_001.pdf]

Supplemental Information:

Second-order Kinetic Rate Coefficients for the Aqueous-phase Sulfate Radical ($SO_4^{\cdot -}$) Oxidation of Some Atmospherically Relevant Organic Compounds

Lillian N. Tran,[1] Karizza A. Abellar,[2] James D. Cope,[1] and Tran B. Nguyen[1]*

1. Department of Environmental Toxicology, University of California Davis, Davis CA 95616, USA
2. Department of Chemistry, University of California Davis, Davis CA 95616, USA

Table S1. Estimation of the competition between the reaction of SO_4 and OH with organic compounds in this work.

**Entity**	**Value**	**Units**	Calculation or Notes
$K_2S_2O_8$	0.02	M	
Erythritol	0.005	M	
J_{S2O82-}	5.50E-05	s^{-1}	Empirical
$[SO_4^{\cdot-}]$ formation rate	1.10E-06	M/s	$J_{S2O82-}*[S_2O_8^{2-}]$
$k_{SO4+H2O}$	400	s^{-1}	Tang et al., 1988
$k_{SO4+ Eryth}$	4.20E+07	$M^{-1}\ s^{-1}$	Hoffmann et al., 2009
$k_{OH + Eryth}$	1.90E+09	$M^{-1}\ s^{-1}$	Hoffmann et al., 2009
$[SO_4^{\cdot-}]_{ss}$	5.23E-12	M	$J_{S2O82-}*[S_2O_8^{2-}]$ / $(k_{SO4+H2O} + k_{SO4+ Eryth}*[Eryth])$
[OH] formation rate	2.09E-09	M/s	$k_{SO4+H2O}*[SO_4^{\cdot-}]_{ss}$
$[OH]_{ss}$	2.20E-16	M	$k_{SO4+H2O}*[SO_4^{\cdot-}]_{ss}$ / $(k_{OH + Eryth}*[Eryth])$
**% Erythritol reacting with OH**	**0.19%**		$(k_{OH + Eryth}*[OH]_{ss}*[Eryth])/(k_{OH + Eryth}*[OH]_{ss}*[Eryth] + k_{SO4 + Eryth}*[SO_4^{\cdot-}]_{ss}*[Eryth])$
**% Erythritol reacting with $SO_4^{\cdot-}$**	**99.81%**		$(k_{SO4 + Eryth}*[SO_4^{\cdot-}]_{ss}*[Eryth])/(k_{OH + Eryth}*[OH]_{ss}*[Eryth] + k_{SO4 + Eryth}*[SO_4^{\cdot-}]_{ss}*[Eryth])$

Table S2. Direct photolysis coefficients (J) for select compounds of interest under the 254 nm UV lamp used in the study. Estimated quantum yields of chromophore loss (ϕ) were extracted over the entire wavelength range of the study.

**Compound**	**J (s^{-1})**	$\Phi_{-\ chrom.}$
Pyruvic Acid (pH 2)	4.05×10^{-4}	2.4
Pyruvic Acid (pH 5)	4.67×10^{-5}	0.19
MDDN	5.07×10^{-5}	0.22
MT3N	5.08×10^{-5}	0.06
1,2-DHI	1.61×10^{-4}	3.2

Section S1: The photolysis correction follows from the mathematics of the pseudo first order kinetics, where k is the second order rate coefficient for erythritol (E) and the compound of interest (A), the effective rates are denoted k' (= $k[SO_4^{\cdot -}]_{ss}$), and J is the first order photolytic rate coefficient for A in the photolysis control experiments (**Table S2** and **Fig. S3**).

$$E = E_0 \exp\left(-k'_E * t\right)$$
$$A = A_0 \exp\left(-(k'_A + J) * t\right)$$

Therefore:

$$\ln\left(\frac{E_0}{E}\right) = k'_E * t$$
$$\ln\left(\frac{A_0}{A}\right) = (k'_A + J) * t$$

A ratio of k_E/k_A is achieved by:

$$\frac{k_E}{k_A} = \frac{k_E[SO_4^{\cdot -}]}{k_A[SO_4^{\cdot -}]} = \frac{k'_E}{k'_A} = \frac{\ln\left(\frac{E_0}{E}\right)}{\ln\left(\frac{A_0}{A}\right) - J * t}$$

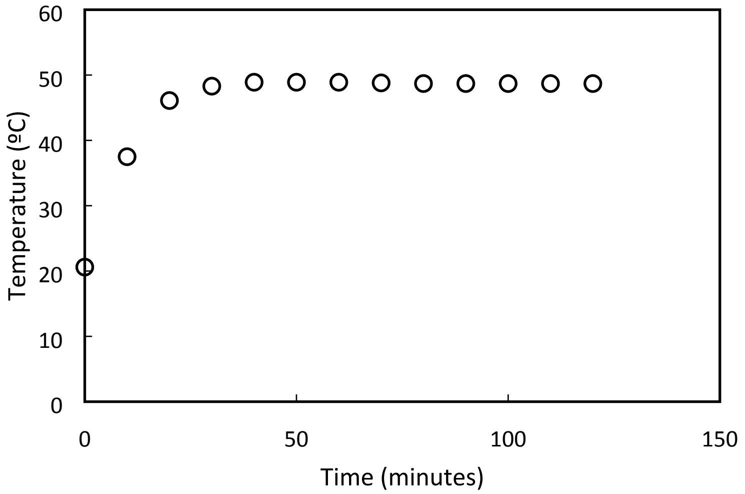

Figure S1 Temperature of photooxidation chamber over the course of 2 hours.

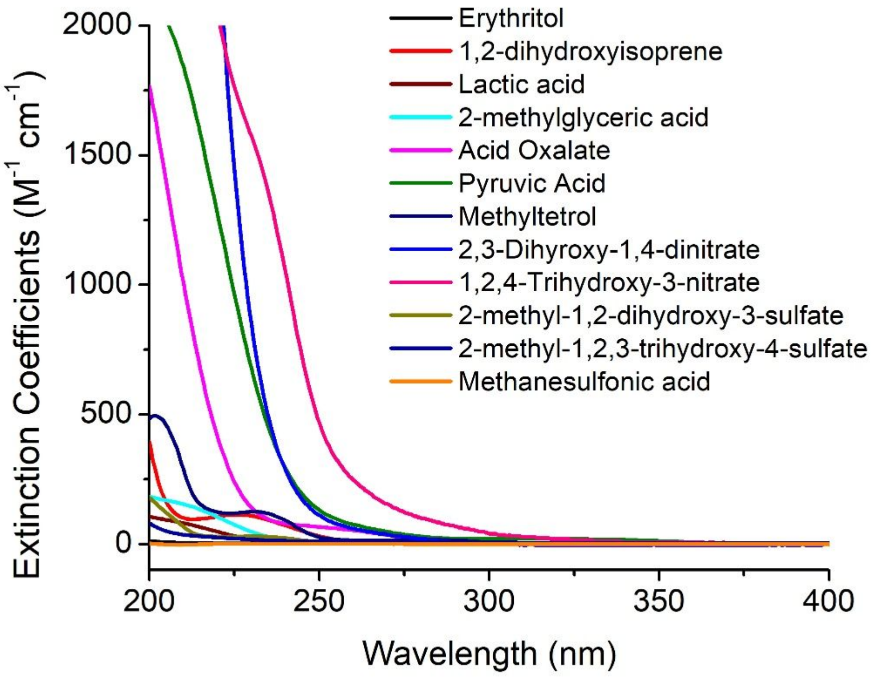

Figure S2 A magnified view of Figure 3b in the main text. Note that the 2-methyltetrol has a small absorption in the 200-250 nm range that is not expected based on its functional groups. Its absorbance is expected to be similar to erythritol, as the only difference is a methyl group. This optical artifact is likely due to small impurities from the synthesis and purification procedure of methyltetrol that is not expected to affect the kinetic determinations.

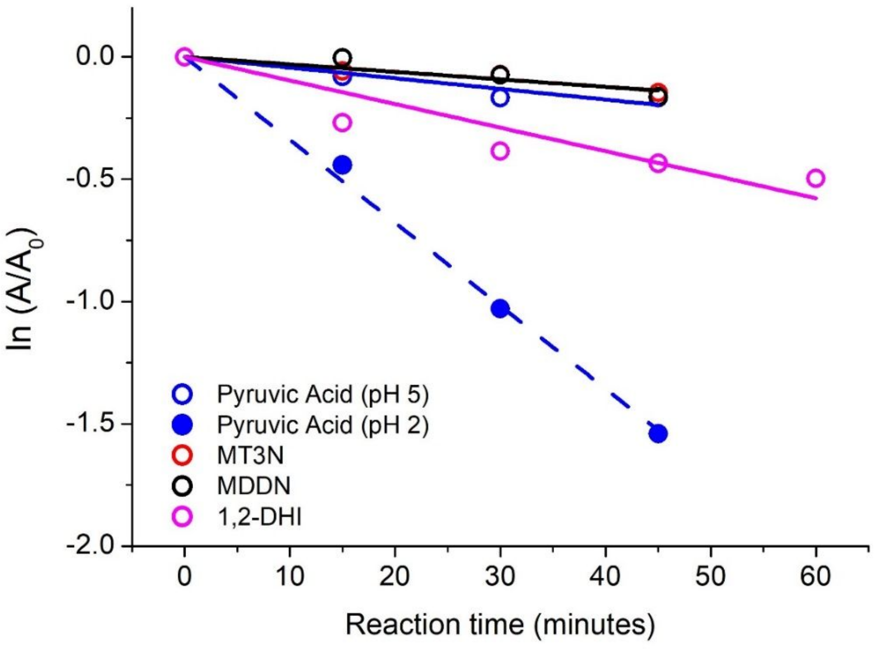

Figure S3. Direct photolysis results for select compounds under 254 nm UV light in our experimental apparatus.

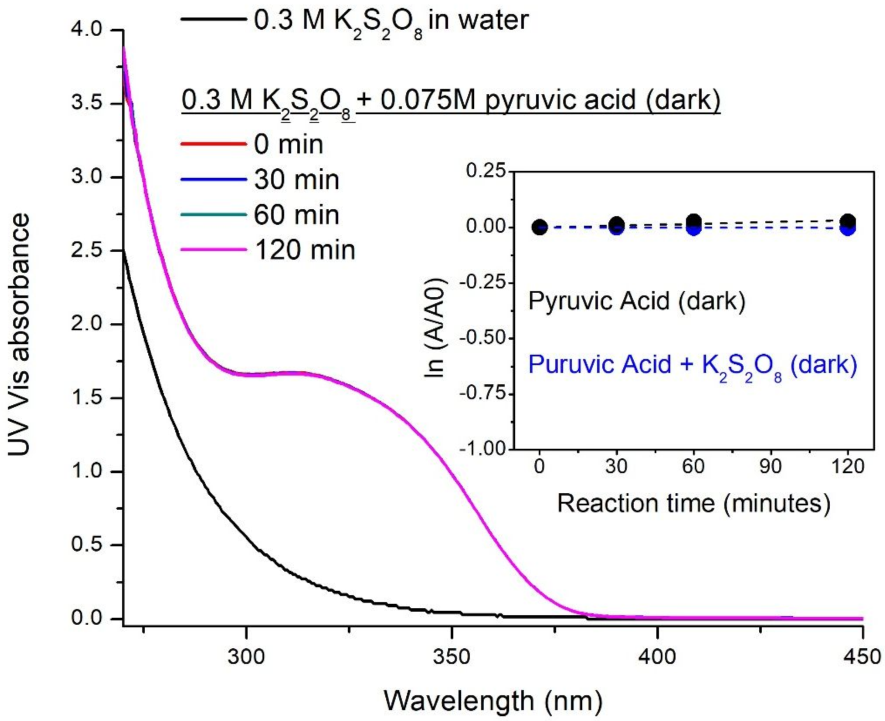

Figure S4. Dark control of $K_2S_2O_8$ with pyruvic acid, as monitored by UV-visible spectroscopy. The insert shows the kinetic decay of pyruvic acid (monitored at 360 nm) by itself in water and when mixed with 0.3M of $K_2S_2O_8$ in water. It was concluded based on the control studies that the $K_2S_2O_8$ itself does not react with the organics at the concentrations used in the study.
